# Causes of Non-Adherence to Allergen-Specific Immunotherapy: A Foundation towards a Patient-Personalized Approach

**DOI:** 10.3390/jpm13081206

**Published:** 2023-07-29

**Authors:** Erminia Ridolo, Francesca Nicoletta, Alessandro Barone, Martina Ottoni, Gianenrico Senna, Giorgio Walter Canonica

**Affiliations:** 1 Allergology Unit, Parma University Hospital, 43121 Parma, Italy; 2 Department of Medicine and Surgery, University of Parma, 43121 Parma, Italy; 3 Allergy Unit, Verona University Hospital, 37134 Verona, Italy; 4 Personalized Medicine, Asthma and Allergy, IRCCS Humanitas Research Hospital, Via Manzoni 56, Rozzano, 20089 Milan, Italy; 5 Department of Biomedical Sciences, Humanitas University, Via Rita Levi Montalcini 4, Pieve Emanuele, 20072 Milan, Italy

**Keywords:** allergen immunotherapy, adherence, persistence, personalized therapy, subcutaneous immunotherapy, sublingual immunotherapy

## Abstract

Background. Allergen-specific immunotherapy (AIT) is the only disease-modifying therapy for allergic conditions, resulting in a long-lasting tolerance beyond the duration of the treatment. Due to the strong relationship between the effectiveness, its optimal duration (at least three years) and the observation of the correct administration protocol, appropriate adherence to the plan of treatment represents a critical factor for the therapeutical success of AIT. Methods. Analysis of studies about the rate of adherence in subcutaneous and sublingual immunotherapy, which are the main routes of administration of AIT. Results. There are different causes leading to a premature interruption of the therapy or to it being incorrectly carried out; the most reported include erroneous expectations of the effectiveness and the adverse effects, economic issues, inconvenience and unrelated clinical conditions. Conclusions. An attentive analysis of the main causes of dropouts may be useful to improve the management of these patients and to develop new strategies for a personalized approach. These strategies should be dynamic, involving attentive communication between the physician and the patient about all the possible criticalities, especially in the initial phase of the therapy, and facilitating, as much as possible, access to healthcare providers over the course of the maintenance phase, including by exploiting technological tools.

## 1. Introduction

Amongst all of the treatments aimed at controlling hypersensitivity to different types of allergens (e.g., Hymenoptera venom, inhalants, etc.), allergen-specific immunotherapy (AIT) stands out, due to its peculiarity to promote a regulation of T helper 2 response, which makes it the only disease-modifying therapy for allergic conditions. In contrast to antihistamines and steroids that ensure, at most, symptomatic control of the allergy, AIT provides a long-lasting tolerance, persisting beyond the duration of the treatment, and the prevention of the development of new different allergies [1]. It is also commonly known, however, that, in order to make it effective and to provide beneficial long-term effects, patients have to exhibit protocol adherence for at least three years to both subcutaneous (SCIT) and sublingual (SLIT) immunotherapy, which are the main administration routes for AIT [2]. For this reason, allergists constantly have to face the challenge of keeping patients well-motivated for a very long time, taking into account the possible causes of non-adherence, such as ineffectiveness, side effects, costs and further unsuspected factors. In order not to cause a premature ending of what should be a long relationship between the allergist and the allergic patient, every strategy which can usefully provide a personalized treatment can make the difference between an effective or an ineffective approach. In this scenario, a critical excursus of the most frequent causes of non-adherence may prove helpful for the management of the patients who undergo AIT, providing food for thought on what can be improved. 

## 2. Definition of Adherence, Compliance and Persistence

According to the World Health Organization (WHO), the term “adherence” refers to the extent to which a patient’s behavior, such as taking medications, following a diet or changing their lifestyle, corresponds with agreed recommendations from a healthcare provider [3]. Particularly, this term includes the concept of persistence, which may be defined as the period from the initiation of the AIT to the last dose before the discontinuation [4], which should idealistically persist at least three years [5]. Moreover, adherence is more appropriate to describe the crucialness of the involvement and the awareness of the patient than the term “compliance”, which instead refers to a more passive role of the patient, leaving to the physician all the decisions about treatment plans [6]. Adherence may, hence, be conceived as persistence in accordance with the schedule of AIT [4], and, for this reason, another key point is the choice of the schedule of treatment. 

Concerning the measurements of the rate of adherence, the situation appears very heterogeneous, highlighting the lack of a defined gold standard. Direct methods, such as the direct observation or the measurement of the level of medicine in the blood, are difficult to practice in real life. On the other hand, indirect methods are easier to obtain, such as patients’ self-reports, pill counts or rates of prescription refill, but these may imply an intrinsic bias (higher awareness of the patient) or may be easily distorted by patients, especially in the case of SLIT [6]. As shown in Table 1 and Table 2, this heterogeneity partly contributes to the broad discrepancy reported between the trials carried out using SCIT (ranging from 16% to 95%) and SLIT (ranging from 5.2% to 78%) in the last years. This variety in results in the literature can also be explained by the differences existing among countries, the composition of allergen vaccines, the treatment schedules and immunotherapy cost and funding [7]. 

Even in the absence of an official consensus on the rate of adherence for which to strive, most of allergists agree on considering e a rate of at least 80% as acceptable [8]. 

## 3. Main Reported Causes of Dropouts

In order to facilitate the analysis of all the causes of dropouts described in the trials, beyond only the rate of adherence in patients undergoing SCIT and SLIT, Table 1 and Table 2 provide also, whenever possible, a framework of the rates related to the categories of factors causing the dropouts. Based on the available data in the preceding tables, Table 3 reports the average rate for the different causes of dropouts. Due to the limited data (especially for SLIT) and to the heterogeneity of methods and populations of the different studies considered, the aim of this table is only to show an indicative picture about the reasons for discontinuation of AIT. Further studies not affected by this kind of bias may provide greater clarity about this topic in the future.

**Table 1:** Studies on the rate of adherence in subcutaneous immunotherapy (SCIT) for inhalants and related causes of dropouts before the completion of the third year of therapy.

Reference	Sample (n)	Age (Years)	Country	Unspecific Adherenc * at 3rd year (%)	Measurement Methods	Causes of Dropouts (%)	Factors Favoring Adherence
Cohn et al., 1993 [9]	217	Adults	US	50	No renewal in ≥6 months	Inconvenience (45%) Questionable effectiveness (16%) Unrelated medical conditions (10%) Symptomatic improvement (8%) Unknown (21%)	-
Lower et al., 1993 [10]	315	5–18	US	56	Compliance (unspecified)	-	-
Donahue et al., 1999 [11]	384	Any	US	33	**Adherence to the schedule**	-	-
Rhodes, 1999 [12]	1033	>6	US	**88**	Persistence	Inconvenience: - Non-compliance (22%) - “Inconvenience” (16%) Unrelated medical condition (22%) Moved residence (19%) Adverse reactions (11%) Financial issue (6%) Questionable effectiveness (4%)	-
Pajno et al., 2005 [13]	1886	6–15	IT	**89**	Compliance (unspecified)	Inconvenience: - Time consuming (24%) - Family problems (14%) - Unpleasant (9%) Financial issue (40%) Questionable effectiveness (13%)	-
Hankin et al., 2008 [14]	520	<18	US	16	Persistence	-	-
Hsu et al., 2012 [15]	139	8–82	US	55	Persistence	Inconvenience (65%) Unrelated medical issue (10%) Financial issue (10%) Questionable effectiveness (10%) Adverse reaction (5%)	-
Guenechea-Sola et al., 2013 [16]	156	>18	US	63	>50% of the recommended number of injections	-	Age > 66
Kiel et al., 2013 [17]	2796	Any	NL	23	Persistence	-	General practitioners
Silva et al., 2014 [18]	122	Any	PT	54	**Adherence to the schedule**	-	-
Gelincik et al., 2017 [19]	204	>18	TR	73	**Adherence to the schedule** (no suspensions >2 months)	-	Female gender
Lemberg et al., 2017 [20]	207	13–89	DE	68	Persistence: - 36 months for perennial - 28 months for pre-(co)seasonal	-	Age > 45
Musa et al., 2017 [21]	150	35 ± 13	KW	59	Persistence	Frequency of injections (82%) Duration of treatment (71%) Commuting (68%) Other commitments (52%) Waiting time (23%) Adverse reactions (23%) Symptomatic improvement (13%) Unrelated clinical conditions (9%) Questionable effectiveness (6%) Traveling (5%)	-
Yang et al., 2018 [22]	311	Any	CH	65	**Adherence to the schedule** (no suspensions for >3 consecutive months)	Inconvenience (33%) Questionable effectiveness (25%) Symptomatic improvement (23%) Adverse reactions (14%) Other reasons (pregnancy, costs) (5%)	Age < 18
Sondermann et al., 2018 [23]	272 (225 SCIT)	6–85	DE	**84**	No suspensions	Time consuming (69%) Adverse reactions (63%) Questionable effectiveness (61%) Insufficient information (54%) Relatives against the therapy (46%) Difficult integration in everyday life (42%)	-
Tat, 2018 [24]	95	17–63	TR	65	**Adherence to the schedule**	Financial issue (33%) Inconvenience: - Inconvenience (15%) - Family problems (7%) Questionable effectiveness (15%) Moved residence (7%) Unrelated medical conditions (pregnancy) (11%) Adverse effects (11%)	Monthly household income
Lee et al., 2019 [25]	1162	5–70	KR	**80**	No suspensions	-	> 40 years Female gender Conventional schedule
Borg et al., 2020 [26]	19447	Any	DN	57	Purchase data from a national prescription database	-	-
Lourenco et al., 2020 [7]	323	7–65	PT	**84**	Persistence	Financial issue (48%) Questionable effectiveness (23%) Switch to SLIT (12%) Inconvenience (personal issues) (8%) Adverse reactions (4%) Unrelated medical conditions (5%)	-
Pfaar et al., 2023 [27]	71085	>5	DE	6.4–18.5 **	Purchase data from a national prescription database	-	-

* Due to the misconceptions encountered in several studies among the definitions of “adherence”, “persistence” and “compliance”, for reasons of simplification, all three eventualities are named as “adherence”, reporting the specific measurement method for each study. ** Due to the lack of a cumulative rate of adherence, instead of being categorized in 6 groups (“Depigmented polymerized allergen extract” and “other subcutaneous AIT” in three allergen groups—grass pollen, early flowering tree pollen and house dust mites), a range of rates has been reported.

**Table 2:** Studies on the rate of adherence in sublingual immunotherapy (SLIT) for inhalants and related causes of dropouts before the completion of the third year of therapy.

Study	Sample (n)	Age (Years)	Country	Adherence * at 3rd Year (%)	Measurement Method	Causes of Dropouts (%)	Factors Favoring Adherence
Pajno et al., 2005 [13]	806	6–15	IT	78	Compliance (unspecified)	Inconvenience: - Time consuming (17.9%) - Family problems (15.0%) - Unpleasant (5.8%) Financial issue (36.4%) Questionable effectiveness (24.9%)	-
Hsu et al., 2012 [15]	78	8–82	US	59	Number of SLIT vials received y the patient	Questionable effectiveness (50%) Financial issue (38%) Adverse reactions (12%)	-
Kiel et al., 2013 [17]	3690	Any	NL	7	Persistence	-	-
Lemberg et al., 2017 [20]	123	13–89	DE	61	Persistence: - 36 months for perennial - 28 months for pre-(co)seasonal	-	Age > 45
Musa et al., 2017 [21]	86	35 ± 13	KW	12	Persistence	Inconvenience (43%) Duration of the therapy (13%) Traveling (12%) Adverse reactions (35%) Symptomatic improvement (30%) Questionable effectiveness (25%) Unrelated clinical condition (4%)	-
Borg et al., 2020 [26]	2471	Any	DK	53	Purchase data from a national prescription database	-	-
Kikkawa et al., 2023 [28]	53	34–62	JP	64 (at 26 ± 11 months)	**Adherence to the schedule**	-	Age > 40
Pfaar et al., 2023 [27]	32543	>5	DE	5.2–7.6 **	Purchase data from a national prescription database	-	-

* Due to the misconceptions encountered in several studies among the definitions of “adherence”, “persistence” and “compliance”, for reasons of simplification, all three eventualities are named as “adherence”, reporting the specific measurement method for each study. ** Due to the lack of a cumulative rate of adherence, instead of being categorized in 6 groups (“Depigmented polymerized allergen extract” and “other subcutaneous AIT” in three allergen groups—grass pollen, early flowering tree pollen and house dust mites), a range of rates has been reported.

It is necessary to specify that, due to the misconceptions encountered in several studies among the definitions of “adherence”, “persistence” and “compliance”, for reasons of simplification, all three eventualities are reported in the tables as “unspecific adherence” while detailing the specific measurement method used in each study. Hence, the unspecific adherence rate at the third year of SCIT ranges from 6.4 to 89%, but, considering the purest meaning of adherence (comprising an acceptable fulfilment of the schedule treatment and the persistence for at least three years), a lower heterogeneity was reported, ranging from 33% to 73%. Regarding SLIT, the unspecific adherence rate ranged from 5.2 to 78%, with only one study reporting a rate of adherence to the schedule of 64%. 

### 3.1. Erroneous Expectations of the Effectiveness of Therapy

Unrealistic expectations about the effectiveness of AIT, in both the negative and the excessively optimistic sense, represent a considerable part of the reasons for non-adherence. The most recurrent issue among the causes of discontinuation of AIT may be categorized as “questionable effectiveness” [7,9,12,13,15,21,22,23,24], which, aside from the purely pessimistic ones, basically involves the patients who expected to experience a greater benefit from the use of drugs (i.e., antihistamines). Particularly, the study of Yang et al. [22] described doubtful effectiveness as the leading cause of dropouts for children, who, moreover, reported a rate of adherence higher than adults. This may suggest that, in matters of children’s health status, parents usually pay more attention than in that of their own, being more likely to eventually sacrifice money and time if they deem it clinically advantageous. In this regard, careful education of patients (or parents of the patients) is of the utmost importance for preparing them to not expect an immediate clinical improvement. 

Taking also into consideration that patients who adhere to immunotherapy in the first year are more likely to complete it [7], providing clear information about goals, risks and appropriate duration of AIT prior to its prescription may turn out to be effective [20]. Particularly, an educational program involving patients being treated with SLIT, executed through an appropriate dedicated time to provide information on allergic rhinits, practical performance, optimal length of treatment and possible side-effects, such as via the delivery of written instructions instead of only verbal advice, showed an increase in the adherence of patients [29]. In this respect, simple measures such as providing a template with a clear and concise summarization of the information on the characteristics of allergic disease and the features and mode of action of AIT may be helpful [30].

This could be achievable, not only in the cases of eventual non-effectiveness in the first periods, but, paradoxically, also in the cases of early perception of symptomatic improvement, which is, on the other hand, another relevant cause of interruption of AIT, due to the patient feeling they no longer need the treatment once the symptoms are controlled [9,21,22]. 

In addition, along the theme of expectations, a further aspect must be evaluated, that is, the clinical condition leading to the initiation of AIT. In this respect, it is not surprising that, while the trials regarding AIT for inhalants very rarely achieved a rate of adherence higher than 80%, in the study of Bilò et al. [31] concerning SCIT for Hymenoptera venom, a rate of adherence of 95% was stated, probably due to patients’ self-motivation due to their perception of an unpredictable life-threatening risk.

### 3.2. Adverse Reactions

Adverse reactions represent another main class of the reasons for dropout from AIT. It is known that SCIT is related to a higher number of systemic adverse reactions, with a risk of fatal or near-fatal reaction being minimal but not absent [29]. Although SLIT application causes systemic side effects in only 1% of cases, an earlier and higher dropout has been reported in patients treated with SLIT than in the ones treated with SCIT [27]. This may be caused by a higher rate of milder but discomforting side effects (approximately 80–90%) especially during the first weeks, such as oral mucosal pruritus (particularly in patients with pollen–food syndrome), mild swelling of the tongue or gastric discomfort (e.g., vomiting, abdominal pain and diarrhea) [29]. To avoid evitable interruptions, easy and fast feedback by physicians, even by phone calls, instant messages or emails, could prove useful [22]. 

To mitigate the risk of severe systemic reactions, a prior stratification of such a risk with appropriate adjustments in patient selection (e.g., excluding patients with uncontrolled asthma or a history of prior severe reaction), standardization of immunotherapy extracts and improving protocols (e.g., by reducing the doses of vaccine during the seasonal pollen peak, avoiding rush build-up protocols when inappropriate or using pre-medications), adequate post-administration observation and informed consent represent a good practice to prepare the patient and to reduce or adequately manage such undesirable occurrences [12,29]. 

### 3.3. Unrelated Medical Conditions

Sometimes, the adherence to AIT may be affected by the unexpected onset of an unrelated clinical condition, leading the patient to leave the treatment, even if not strictly necessary. In terms of contraindications to AIT, autoimmune disorders and cancer, particularly in active phases, have been usually considered “absolute” or “relative” contraindications for the initiation or the maintenance of AIT [32]. In particular, regarding malignancies, even if an active cancer currently represents a relative contraindication, a past cancer would not be contraindicated [33]. 

A more emblematic example of unnecessary interruption of AIT is the case of pregnancy, frequently reported as a cause of dropout [22,24]. It is estimated that approximately one-third of women experience a worsening of respiratory symptoms during pregnancy [34], with 20% experiencing an asthmatic exacerbation [35], but, despite this, the concerns about eventual repercussions on the unborn child often overcome the needs of the expectant mother, even if it paradoxically leads to an increase in the risk of hospitalization. In addition, even the risk of life-threatening reactions related to Hymenoptera venom allergy should not be underestimated [34]. However, due to the higher risk of near-fatal reactions, in the cases of previous systemic reactions to AIT or in the outbreak of uncontrolled asthma, these concerns are far from unmotivated [36]. In actuality, there are still limited data about the response of the offspring in pregnancies exposed to AIT. A recent study on a Swedish cohort of pregnancies exposed to both SCIT and SLIT found no elevated risk for congenital malformations or other adverse outcomes [37]. Moreover, the initiation of AIT during pregnancy has also been reported to be safe, both in a retrospective study for SCIT [38] and in a prospective study for SLIT [39], though it is necessary to point out the limited number of cases considered. Relying on a larger number, an allergists’ survey of the American Academy of Allergy, Asthma & Immunology, with respect to SCIT, highlighted a substantial agreement on the continuation of the immunotherapy during the pregnancy, contrasting with the initiation, which is considered (at best) a relative contraindication [40]. To summarize, in pregnancy, more than in other conditions, a shared decision-making process, with the allergist providing explanations about actual and unfounded reservations, is advisable, for the correct management of the patient and to solidify the doctor–patient relationship [41].

Furthermore, the psychiatric condition of the patient must be evaluated before the prescription of AIT, e.g., anxiety and panic disorder may be described as predictable warning signs for early dropout [12]; as well, those with needle phobia [33] should be directed to the choice of SLIT rather than SCIT. On the other hand, retrospective studies conducted on veterans in the Los Angeles and Memphis areas both highlighted an increased adherence in patients affected by posttraumatic stress disorder (PTSD), probably because of their regular physician visits and the absence of employment commitments, as they were considered legally disabled [16,42].

### 3.4. Financial Issues

A different category of causes of early interruption of AIT relates to the financial aspects, involving the costs of the extracts, the visits and the co-payment for each injection in the case of SCIT [11], the loss of working hours/days or the dependence on the benefit of reimbursements or insurance coverage that, most of the time, are penalized by slow bureaucracy [22]. Clearly, much depends on the country of residence and its healthcare system, as well as on social factors. In a Turkish study, in fact, adherence was favored in a significant way by a higher monthly household income than those below the poverty threshold for a family of four people [24]. On the contrary, the results of a US trial suggested that cost was not a relevant factor, reporting that paying patients were more adherent than those covered by medical assistance [10].

Given this, in the prescription phase of AIT, the economic possibilities should always be considered and discussed with the patient, with particular attention given to eventual reimbursement; for example, in some regions of Italy, some types of SLIT are reimbursed, which is the opposite of SCIT. Another aspect not easily understood by patients, but worthy of note, is the significant reduction in the use of symptom-relieving medications, that, in the long term, may result in equal or even higher costs than AIT [43]. In a systematic review, in fact, economic modelling found that, when compared with symptomatic treatment, both SCIT and SLIT may become cost-effective per quality-adjusted life-year after around 6 years [44]. 

In addition, in order to promote adherence throughout the course of the therapy, it would be appropriate to reduce to the indispensable minimum the number of visits and to allow patients as many access times as possible, avoiding the interference with their working hours and favoring, when possible, a shift from SCIT to SLIT whenever a patient misses too many appointments because of work.

### 3.5. Inconvenience

Although the term “inconvenience” is listed nearly always among the first reasons for interruption of AIT, representing 15–65% of the causes of discontinuation in SCIT, the meaning attributed to it in different trials is heterogeneous, incorporating different elements. On the whole, inconvenience could be described as a non-convenient relationship between the investment of money, time, effort and compliance and the symptomatic improvement. Hence, the perception of convenience can vary a lot based on the initial clinical condition. For example, in the context of respiratory allergic pathologies, asthmatic patients, whose conditions significantly interfere with their daily activities, report a rate of adherence higher than patients with allergic rhinitis [7]. In addition, a study executed in South Korea found a disease duration lower than 5 years to be a non-adherent factor, probably because patients affected by shorter disease may still not have enough information about the consequences, which is opposite to patients with longer disease, who may have already experienced the lack of efficacy of using only symptomatic medication [25].

Another way to reduce the inconvenience is to minimize, as much as possible, time-consuming situations by providing a direct phone number and a schedule with extended hours in order to offer several options for SCIT administration [7]. As proof of that, a retrospective trial conducted in Netherlands, where both medical specialists and general practitioners can initiate SCIT, found the prescriber as an independent predictor of persistence. In contrast to expectations, though, persistence was higher in patients treated by general practitioners, probably due to the proximity and easy accessibility [17]. Moreover, another problem reported is related to the periodic renewal of AIT. An Italian study on patients undergoing SLIT for seasonal and perennial respiratory allergens showed how the presence of trained administrative staff in charge of contacting patients for the renewal of AIT and facilitating the communication with the allergist for any medical problems or adverse reactions to AIT led to an increase in the renewals [45]. 

Furthermore, accelerated schedules may be considered for patients with active social performance [25], as well as, in particular cases, a longer period between the injections (3 months versus 2) in maintenance of SCIT for Hymenoptera venom, preserving, in this way, safety and efficacy, and improving adherence [46]. This was confirmed in a COVID-19 post-pandemic study, in which was recommended more caution in resuming immunotherapy in patients with long pre-pandemic maintenance intervals, severe pre-immunotherapy reactions, recent initiation, older age, multidrug treatments and bee venom allergy [47].

## 4. The Use of Technology to Improve the Adherence to AIT

The widespread use of telemedicine, digital health tools and apps—reported even more after the coronavirus pandemic—may turn out to be a formidable ally to accomplish the goal of increasing the adherence of allergic patients to AIT, which is especially important for those living in rural or remote areas. 

Reminders of administration generated by digital applications may help to avoid the loss of the number of patients who do not correctly adhere just because of forgetfulness, especially in the case of SLIT; as well, digital diaries that are shared with the allergist may provide a prompt modification of the therapy regimen in response to a symptomatic amelioration or worsening, as well as the onset of adverse effects [39]. Obviously, for those who are not confident with technology, the same objectives can be achieved through phone calls. 

In this context, to ensure patient-centeredness in the elaboration of new applications in the scope of telemonitoring, developing care pathways and clinical decision support systems, patients and allergists should be involved in every step of the design, implementation and update process. Patient support programs could be useful to integrate and optimize communication, educational, motivational and behavioral aspects [48].

In terms of scientific investigation, the collection of a big dataset, characterized by a greater homogeneity of measurements, could surely be helpful for the identification of still unmet needs, so as to enable prospective and pharmaco-economic evaluations in real-life populations [48].

## 5. Conclusions

**Figure 1:**
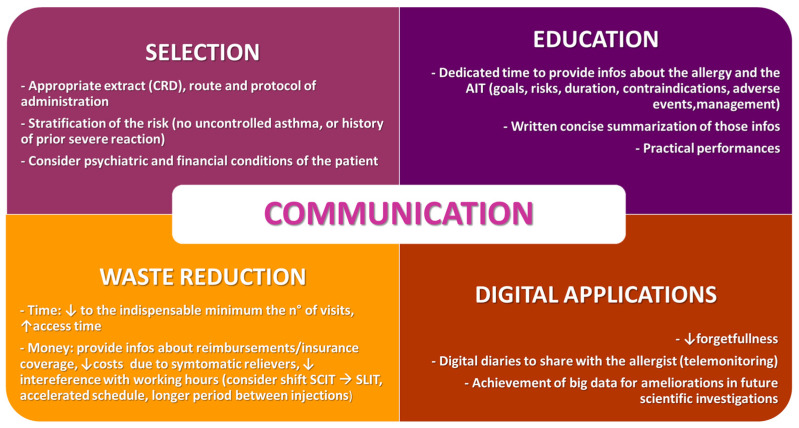
Tips to improve the adherence to AIT.

The effectiveness of long-term treatments such as allergen immunotherapy is strictly influenced by the adherence of the patient, which should never be taken for granted. In Figure 1, some tips to improve the adherence to AIT are listed. 

Currently, the rate of adherence and persistence are far from the optimum, especially for respiratory allergies; thus, a tailored and dynamic strategy may, in the end, provide considerable advantages in terms of clinical outcome. In this scenario, precise and easy communication shows itself to be of the utmost importance to avoid erroneous expectations about the effectiveness of AIT, concerning both treatment time and adverse reactions. Moreover, the economic aspect always has to be considered in its entirety, considering the cost of the extracts and of the eventual injections (in the case of SCIT), the possibility of insurance coverage or reimbursement and the loss of working hours. In this regard, an attentive schedule of the appointments and the possibility to choose the time of the visit, along with the use of technological tools, may be helpful to increase patients’ adherence. However, wide heterogeneity affects the studies conducted until now, above all because of the differences among countries and measurement methods. For all these reasons, more studies in real life should be conducted, to highlight unmet needs and to elucidate even more personalized strategies. 

## Figures and Tables

**Table 3:** Average rates for the different causes of dropouts. Due to the limited data (especially for SLIT), and to the heterogeneity of methods and populations of the different studies considered, the aim of this table is only to show an indicative picture of the reasons of discontinuation AIT. *p*-values have been calculated by employing the chi-square test.

Causes of Dropout	Average Rate in SCIT			Average Rate in SLIT			*p*-Value
	References Reporting the Rate of That Cause	Cumulative Population	%	References Reporting the Rate of That Cause	Cumulative Population	%	
Inconvenience	[7,9,12,13,15,22,24]	1614/4004	**40**	[13,21]	349/892	**39**	0.514028
Financial issues	[7,12,13,15,24]	1018/3476	**29**	[13,15]	323/884	**36**	0.00003
Adverse effects	[7,12,15,21,22,23,24]	393/2323	**17**	[15,21]	39/164	**24**	0.024963
Questionable effectiveness	[7,9,12,13,15,21,22,23,24]	676/4426	**15**	[13,15,21]	261/960	**27**	<0.00001
Unrelated medical conditions	[7,9,12,15,21,24]	303/1957	**11**	[21]	3/86	**4**	0.002283
Symptomatic improvement	[9,21,22]	108/678	**6**	[21]	26/86	**30**	0.001017
